# “They Stay With You”: Nursing Home Staff's Emotional Experiences of Being in a Close Relationship With a Resident in Long-Term Care who Died

**DOI:** 10.1177/08980101211017766

**Published:** 2021-05-28

**Authors:** Anne Kristine Ådland, Birgitta H. Gripsrud, Marta H. Lavik, Ellen Ramvi

**Affiliations:** 56627 University of Stavanger; The Research Group for Nursing- and Healthcare Science, 60496 Stavanger University Hospital; 87446 VID Specialized University; Stellenbosch University; 56627 University of Stavanger; 56627 University of Stavanger

**Keywords:** nursing homes, long-term care, holistic nursing, professional–patient relationship, death, emotions, ethics

## Abstract

**Aim:** To explore and develop understanding of nursing home staff's emotional experiences of being in a close relationship with a resident in long-term care who later died. **Design:** Ethnographic fieldwork. **Methods:** As part of fieldwork, narrative interviews were conducted with nursing home staff (*n* = 6) in two nursing homes in Norway and analyzed using interpretative phenomenological analysis. **Findings:** Through data analysis, we identified three superordinate themes: (1) wanting to be something good for the resident and their families, (2) striving to make sense of the resident's death, and (3) struggling to balance being personal and professional. **Implications for holistic nursing and conclusion:** Nursing home staff experience tensions between ideals of distanced professionalism and the emotional experience of proximity, evidenced by personal commitment and mutual recognition in relationships with “special residents” in long-term care. To support holistic practice, awareness is needed of the emotional impact of relationships on health professionals. Suppressing feelings puts staff at risk of moral distress, compassion fatigue, and burnout, as well as higher turnover and absenteeism. Managers should facilitate discussions on professionals’ ideals of relationship-based practice, including processing of, and reflection on, emotional experiences in long-term care. Rituals to mark a resident's death can provide further emotional containment.

## Introduction

This article concerns nursing home (NH) staff's emotional experiences of being in a close relationship with a resident who later died. The present study evolved as part of an ethnographic fieldwork (Ådland et al., 2019) on how health professionals in multicultural staff communities encounter death in NHs, and how institutional procedures, structures, and rituals support or undermine communication about their personal and existential experiences.

A striking experience during the fieldwork was the observation that NH staff seemed to establish very close relationships with residents. The following two fieldnote excerpts are illustrative vignettes of this observation.

Excerpt one:

I’m sitting in the living room together with staff and residents. One of the residents has been asking for health care worker ‘Timmy’ several times today and when Timmy enters the living room, the resident clearly becomes happy. His face lights up and he immediately asks Timmy to follow him to his room. Timmy smiles at him and I sense the warmth between them. With a cheerful voice, Timmy says “of course, come on.” The resident is very frail, and Timmy finds the resident's walker and helps him up from the couch. They walk slowly down the corridor while Timmy is holding his arm and I can hear them laughing.

The first author noticed that Timmy often sat with this resident, talking to him, and helping him, and also heard several times that the resident asked for Timmy when he was off work. The impression was that the two of them had a very close relationship.

Excerpt two:

A vintage car parade is coming to the nursing home today and the staff are helping residents to go outside to see the parade. The nurse ‘Reidun’ tells me that one of the residents she cares for, who is terminally ill, has a passionate interest in vintage cars. Reidun has cared for the resident over a long period, and she knows it will be a great experience for him to have one last ride in a vintage car, she says. Together with the resident's son, she has arranged for such a ride today with an owner of a vintage car, and when the son arrives at the nursing home, I accompany them to the resident's room. The resident is very weak and emaciated. Reidun and the son have to lift the resident into a wheelchair. I follow them down to the front door. The vintage car is standing right outside, and together with the driver, they manage to lift the resident into the backseat of the car. Reidun and the son place themselves one on each side of the resident. As they drive, I can see them all smiling having tears in their eyes. For me, it is a very touching moment to witness.

Based on these and several other observations from the fieldwork, we became curious to know more about how NH staff experience being in close relationships with long-term care residents who later died. We therefore decided to pursue this further by conducting narrative interviews to gain more insight into the health professionals’ emotional experience of these relationships.

## Background and Significance

In Norway, over 50% of all registered deaths occur in NHs (Norwegian Institute of Public Health, 2018). Health professionals, by which we mean registered nurses and regulated health care workers who work in NHs, therefore regularly encounter dying and death. With an increasingly aging demographic, NHs will continue to provide essential whole person care to long-term residents who sooner or later will die (Norwegian Ministry of Health & Care Services, 2020). Because Norwegian NHs are multicultural workplaces (Norwegian Official Report (NOU), 2017: 2), they offer valuable sites for ethnographic study of broader dynamics of inclusion and marginalization, as well as cultural and professional practices in whole person care for long-term residents.

Previous studies have identified how NH staff can form strong bonds with residents and their families. McCallin (2011, p. 2331) found that nurses caring for patients at the end of life went beyond the “efficiency-effectiveness rhetoric to engage in meaningful ‘being with’ relationships” with patients, thereby emphasizing moral responsibilities in the patient–provider relationship. Staff who engage with residents in NHs therefore likely to experience suffering, loss, grief, and bereavement when the residents die (Boerner et al., 2015; Cagle et al., 2017; Khalaf et al., 2018; Marcella & Kelley, 2015; Simard, 2020). Van Riesenbeck et al. (2015) found that a resident's death may be perceived by care providers as akin to the loss of a loved one and that they may feel emotionally unprepared for it. “Family” is a salient metaphor that some staff use to denote their relationship with residents (Canham et al., 2017; Moss et al., 2003). Another study identified mixed feelings, as staff sometimes referred to death as “liberating,” in the sense that the residents could be released from pain and suffering, while also feeling grief when a resident they knew well has died (Boerner et al., 2015).

Observations from the fieldwork, of which the present study is a part, revealed that NH staff did not talk to each other about such emotional bonds and experiences with residents. There seemed to be a shared attitude that the emotional impact of residents’ deaths could diminish exponentially: that the more frequently staff witnessed death, the less they would feel affected. Such attitudes of “hardening” or emotional distance have previously been found to reinforce an organizational culture of repression and silence around death and dying in NHs (Marcella & Kelley, 2015). It is also reflected in the finding of Österlind et al. (2011, p. 538), who found that in Swedish NHs, “death was held at bay, and the emotions experienced by the staff when an old person died were ignored.” NH staff's emotional experiences with residents’ deaths may affect their ability to provide care (Marcella & Kelley, 2015; Schulz, 2017), and silencing such experiences can adversely affect their ability to continue to provide attuned and adequate holistic care (Ramvi & Gripsrud, 2017). Suppressing feelings, such as grief associated with residents’ dying and death, puts professionals at risk of developing compassion fatigue—“a form of physical, emotional, and spiritual exhaustion that can affect the ability to feel and care for others” and may contribute to higher staff turnover and absenteeism (Schulz, 2017, p. 17).

The literature confirms that NH staff develop close relationships with some residents and may experience significant grief after a death. Further to this, emotional distancing or silencing has been found to be existing but suboptimal strategies to deal with such experiences. Hence, the aim of this study was to explore and develop understanding of NH staff's emotional experiences of being in a close relationship with a long-term resident who later died. The study was guided by the following research question:

What characterizes NH staff's emotional experiences of having been in a close relationship with a resident who later died?

## Methodology

### Design

The overall study design is ethnographic fieldwork (Hammersley & Atkinson, 2007). The ethnographic methodology employs multiple methods of data collection (Hammersley & Atkinson, 2007). Data collection in the fieldwork comprised observations, policy and institutional documents, and field conversations, as well as narrative interviews, which are the main data source for the present study. The interview approach was inspired by the biographical narrative interpretive method (BNIM) (Wengraf, 2001). Interview data underwent an interpretative phenomenological analysis (IPA) (Smith et al., 2009). We opted to use IPA because it allows for a detailed examination of lived experience of key participants in the fieldwork, while also exploring how they make sense of their personal and social world. According to Smith and Osborn (2015, p. 41), IPA is deemed “a particularly useful methodology for examining topics which are complex, ambiguous and emotionally laden,” in line with the scope of this study. The theoretical perspectives that underpin IPA draw on phenomenology, hermeneutics, and idiography. Phenomenologically, IPA is sympathetic to the call for a return to “the things themselves” through a persistent focus on the phenomenon under study, which can illuminate specific qualities of experience. IPA's hermeneutic orientation can be evidenced in its recognition that there is no such thing as an uninterpreted phenomenon (Smith et al., 2009). Central to IPA is therefore a reflexive awareness of the interchange between the participant's narrative and its interpretation, which the researcher brings to the process. Thus, the researcher is engaged in a double hermeneutic “trying to make sense of the participants trying to make sense of what is happening to them” (Smith et al., 2009, p. 3). Finally, IPA's idiographic commitment is evidenced by a detailed examination of the particular case before moving on to analyzing other cases. Only when all cases have been examined separately, can a cross-case analysis occur (Smith et al., 2009).

### Setting and Sample

The fieldwork was conducted in two NH units in Norway over a 6-month period between 2017 and 2018. Residents in these NHs were frail and suffered from cognitive impairment. In ethnography, key participants are individuals with special insight or unique experiences about the research topic or phenomenon under investigation (Hammersley & Atkinson, 2007). During the fieldwork, the first author and field researcher established contact with several of the NH staff who subsequently became key participants. These were staff who actively initiated contact with the field researcher and were eager to share their views and experiences. For the present study, the first author purposively enrolled six key participants (three staff members from each unit) from diverse cultural backgrounds to take part in narrative interviews. We make no knowledge claims here related to the ethnic and cultural diversity of the participants, as this topic is to be explored in a forthcoming publication. The key participants were selected because of their first-hand knowledge and experiences of working with long-term residents in NHs. In ethnographic fieldwork, key participants often constitute a small sample. However, this is not considered a limitation when the content of their narratives is explored in-depth (Hammersley & Atkinson, 2007). Moreover, a small sample is merited by our analytic approach, as the primary concern of an IPA is to develop a detailed account of individual experiences (Smith et al., 2009). This is also in line with Malterud et al. (2016) understanding that sample size in qualitative studies can be guided by the potential for “information power,” and that in-depth analysis of cases require fewer participants than, for example, a thematic cross-case analysis.

Table 1 provides an overview of the characteristics of the participant.

**Table 1.** Participant Characteristics (Gender, Region of Origin, Approximate Age, Years of NH Experience, Education/Qualification)

Pseudonym	Gender	Region of origin	Approximate age	Years of NH experience	Education, qualification
Karina	Female	Central Europe	40s	15-20	Nurse (RN)
Even	Male	Norway	20s	1-5	Nurse (RN)
Kjetil	Male	Norway	30s	5-10	Nurse (RN)
Milana	Female	Central Asia	50s	10-15	Health care worker
Reidun	Female	Norway	40s	10-15	Geriatric nurse
Timmy	Male	Southeast Asia	30s	1-5	Health care worker

### Ethical Considerations

The study was conducted on the principles of protection from harm, informed and voluntary consent, anonymity, and proper data storage (NESH, 2016). Prior to the fieldwork, NHs and participants received written and oral information about the study. Residents and relatives indirectly affected by the fieldwork received an information letter. With varying language proficiency in Norwegian, one challenge was to ascertain whether all participants could comprehend the purpose of the study and what enrolment might entail. To secure informed consent with this aspect in mind, information about the study was repeated, including voluntary participation and the right to withdraw from the study. Participants who agreed to enroll consented in writing on the day of the interview. We have strived to protect the anonymity of the participants, residents, and their families. The names of participants and the NH staff in the vignettes have been changed to protect confidentiality.

### Method for Data Collection

Interviews were carried out by the first author in 2018 and were inspired by the BNIM (Wengraf, 2001). BNIM relies on a special interviewing technique to elicit participants’ uninterrupted stories and thereby capture their way of making sense of their personal, social, and professional worlds (Ramvi, 2015). The BNIM interview was staged in two subsessions. In the first subsession, the interviewer actively listened, inviting uninterrupted narration from the participants and supported their storytelling. Interviews started with a “single question aimed at inducing narrative” (SQUIN) (Wengraf, 2001). In our study, the SQUIN presented by the first author to the six participants was:

As you know, I am interested in how health professionals experience working with people who are going to die. Therefore, can you please tell me about your relationship with a resident you felt you knew well who is now dead? Tell me about all those events and experiences that were important for you personally. Please feel free to take the time you need. I will just listen and won't interrupt you with questions. I will just take notes so that I can remember what I want to ask you about when you have finished telling me about it all. Take the time you need. Start wherever you like.

Subsession two then followed after the participants had told their uninterrupted story in response to the SQUIN. Here the interviewer probed further, asking the interviewee to tell more about items raised in the first subsession, while pushing for more details in particular incident narratives (Wengraf, 2001). In this way, BNIM elicited narration of emotional experience by evoking participants’ memories, rather than their argumentations or justifications (Ramvi, 2015). Unlike a semistructured interview with preset themes, BNIM affords interviewees to decide for themselves what to speak about, and how long they need to tell their stories. Interviews were conducted in the NHs (in a room chosen by the participants), lasting from one and a half to 2 h. Interviews were audiotaped for subsequent verbatim transcription by the first author.

### Data Analysis

We followed IPA's step-by-step guide to develop themes grounded in specific examples from the data set (see Smith et al., 2009, pp. 82–107).

Each interview transcript was analyzed in-depth in four steps. The first step involved the first author getting to know the data material case-by-case, by listening to audio recordings, reading, and rereading transcriptions several times, beginning to reflect. In the second step, line-by-line initial noting was applied consisting of thoughts, possible codes, associations, or connections that came to mind. For each case, a three-column table was designed. Transcribed data were pasted in the middle column, and key points and comments that remained close to the participants’ own words were noted in the left column. In step three, the right-hand column was used to develop emerging themes, and eventually, a summary of emergent themes was created. Step four involved searching for connections across emergent themes, which were then compared with the data and relevant quotes from the transcript were connected with each theme. Themes were arranged and grouped together using color coding. Themes that had weak support in the data set were removed. Steps three and four were experienced as a hermeneutic process of looking at the parts and the whole together, to gain a deeper understanding of the case. Step five of the analysis involved moving on to the next case. The sixth step concerned looking for patterns across cases, which led to the development of superordinate themes across all six cases. The research team created a list to organize superordinate themes and subthemes, including all relevant extracts from each transcript, listed under the appropriate headings. This process involved several revisions, and much careful thought and discussion. Some data extracts were moved from one superordinate theme to another, as part of this concluding process.

After this, transcript excerpts (approximately three pages each) from three of the interviews, which represented different or contradictory cases, as well as the superordinate themes, were presented to two group interpretation panels (for examples of selection criteria, see Smith et al., 2009, pp. 115–6). The strength of an interpretation panel is that it may contribute “to ensure the surfacing of sleeping assumptions, blind spots and misrecognitions on the part of the researcher” (Wengraf, 2001, p. 231), and thereby support thinking together in new and imaginative ways about the data (see also Gripsrud et al., 2018).

### Trustworthiness of the Study

Yardley’s (2000) criteria for evaluating quality in qualitative research were applied to address issues of trustworthiness in this study, and specifically with IPA (Smith, 2011; Smith et al., 2009). The criteria are based on four characteristics: sensitivity to context, commitment and rigor, transparency and coherence, and impact and importance (Yardley, 2000). Sensitivity to context was addressed by relying on “a considerable number of verbatim extracts from the participants’ material to support the argument being made, thus giving participants voice” in the study and allowing our readers to check any knowledge claims made against the data provided (Smith et al., 2009, p. 180). Commitment, rigor, transparency, and coherence relate to expectations of thoroughness in data collection, analysis, and reporting in research (Yardley, 2000). Hence, we have strived to describe the research process in detail, while providing rationale for crucial decisions and actions taken during our work. Although the first author conducted the main work on the IPA case analyses, all authors collaborated on making sense of the data material, providing critical reflection on emergent interpretations and new interpretative ideas, thereby supporting rigor and credibility in the analysis (Smith, 2011; Smith et al., 2009) and the interpretive process (Wengraf, 2001).

Yardley’s (2000) final quality criterion—impact and importance—concerns the ability of a qualitative study to enrich understanding both theoretically and socio-culturally. This has been a key concern in our context-derived study and theoretically informed discussion through which we strive to develop understanding of the emotional impact of close relationships and losses on health professionals working with residents in long-term care. In terms of practical impact and importance, we believe that our findings can contribute to supporting holistic practice and professional wellbeing by articulating and interpreting experiences within long-term care, which is an area in nursing that may benefit from more research-based practice.

## Results

The data analysis led to the development of three superordinate themes, crafted based on emergent subthemes from the case analyses, presented below (see Table 2).

**Table 2.** Superordinate Themes and Subthemes in the IPA Analysis

Superordinate theme	Subtheme
Wanting to be something good for the resident and their families	“We have our own special residents”
	“I give a lot of myself”
Striving to make sense of a resident’s death	“Death is a natural part of life”
	“Have I failed?”
	“It's the best thing for the residents”
	“It's important to say goodbye”
Struggling to balance being personal and professional	“It's not private, you mustn't take it home, but you are a human being”
	“I have to learn to survive”
	“In the end, they are part of our lives in a way”

### Superordinate Theme: Wanting to be Something Good for the Resident and Their Families

#### Subtheme: “We Have our own Special Residents”

In the interviews, participants talk about “special residents” with whom they felt very connected. They describe feeling emotionally close to these residents using words such as “being fond of,” “liking,” and “caring about.” These close relationships are, for several of the participants, defined by reciprocity, being treated with respect, and feeling appreciated. To illustrate, Timmy talks about a special resident he “felt close to and learned a lot from”:

I liked her very much, and I got such comments that when I helped her, she became calm in a way, so when she got upset, they [colleagues] contacted me, and I helped her. So, we somehow became very close. Also, the relatives… when they came, I talked to them and I spent some time with them, and they were as friends…close friends in a way. So, I got to know her and her family very well. […] [When the resident died] I gave a hug to the daughter and son and offered my condolences, and I was a bit tearful…They told me she liked me so well…‘mother liked you’ they said…oh…it was like…and then I couldn't hold back anymore, and I had to cry.

For Timmy, this resident was special to him, and reciprocally he feels that he was special to her and her family. He befriended them, felt valued and able to help, and a bond was established between them.

Other participants refer to their special residents as “close friends” or being “like family,” and having a kin-like family role, with some viewing themselves as an extension of the resident's family. For example, Reidun talks about a resident she felt very connected to:

The one I in a way became most attached to reminds me a lot about my dad… so it was really hard for me (when the resident died). I cried openly…while the relatives were here…and I think they also appreciated it…that someone cared about him. We had good chemistry… he had confidence in me, and I managed to help him with both showering and care without any problems. Many [other colleagues] had problems helping him, they were a little scared of him, and simply didn't dare to come close to him… He had a difficult family situation too; his social network was very small.

Reidun experienced a very strong family-like bond with this resident, and his death therefore made a significant impact on her. The resident had confidence in her and she felt appreciated because this was special; *she* could help him when her colleagues could not.

#### Subtheme: “I Give a lot of Myself”

Knowing that the care they provide has a beneficial impact on the residents is important for the participants. They all emphasize “giving the best care” to residents. For instance, Kjetil said: “I want them to feel good and it’s not just because it’s my duty to do a proper job… it’s because I care. I feel that I care about them.” Other participants talk about “making a difference” through their work and also highlight positive feelings, as they experience a sense of reward in the job when they are able to do something good for residents. The sense of reward is associated with concrete feedback from residents, but can also emerge from *within*, in the sense of feeling fulfillment. As an example, Even talks about a resident he followed over a long period. Having suffered several falls, the resident became bound to the wheelchair. Even dedicated much effort to helping him walk again:

I felt I made a big difference in that resident's life since I made him walk again, twice…and…You can do very few things in a nursing home where you feel you have made a permanent change in a resident's life. Since there is so much going downhill…there is very little going up…but when he first died…. At least it's good to know that you’ve made a difference for that resident.

The participants also talk about striving to do the best they can to care for and comfort when relatives of a dying resident find it difficult to accept the imminence of death: “Sometimes you feel like a psychologist because you are between the two groups, the one who is dying, and those who are standing on the side” (Milana)*.* Participants consider emotional support for relatives important both during and after the dying phase. Participants said they would try to ease the relatives’ fears and anxieties by being available and lending an ear—as is evident in Milana's account where she talks about a relative who strived to accept that his wife was dying:

I sat and talked to him (the dying resident's husband) and he was so grateful. He needed to talk, and he told me that he was so grateful when I was at work. They had been married for over 60 years and it was very hard for him. He even got a hug.

To receive positive feedback is personally gratifying for the participants. Appreciation from residents and their families fosters their feelings of accomplishment and achievement. In turn, this reaffirms their capacity as professional caregivers, enhancing their self-esteem:

I have many good experiences with relatives. They see that I give a lot of myself, being there for both residents and relatives. I’ve received lots of positive feedback, and it makes me feel good also as a person, because then I feel like I’ve done a good job…yes (Reidun).

On the other end of the spectrum, to receive negative feedback can be experienced as undermining of self-esteem. Here, Timmy talks about an interaction with a resident who did not like him:

I wanted to help him, but he didn't like me. I don't know if it was because I’m an immigrant, but every time I tried to help him, he became annoyed and hit me. Of course, I know that as a healthcare professional I have to understand and adapt. I always asked if others could help him, but when nobody else could help, I had to do it. Then he became annoyed and he hit me in both my face and on my body. However, when he got ill, he couldn't do anything to me. Then it felt good…that I finally could do something good for him. I could have said no…that he didn't like me and therefore I wouldn't help him, but I did it anyway…When he died, I remember it was nothing…no feelings.

In this case, Timmy is able to appear professional and caring and feels good about this, but due to their negative prehistory, he has distanced himself emotionally from the resident and feels unaffected by his death.

### Superordinate Theme: Striving to Make Sense of a Resident's Death

#### Subtheme: “Death is a Natural Part of Life”

The participants share the understanding that managing residents’ death is part of their job, a common occurrence in the NH, which they have to “expect” and “accept”:

Entering such a place [nursing home] is really to enter the last place. It's the final station. Many of the residents say that. We just have to accept it in a way…they’ve lived a life. Many of them are old and full of days. (Reidun)

Most of the participants see death as “a natural part of life.” Kjetil for example talks about his first experience with a resident he cared for who died when he was a young student:

When she died, I actually felt a lump in my throat…but I also felt that it would be inappropriate to react and show how upset I was. Therefore, I just had to hold it inside me, but I felt it affected me. At the same time, I thought that of course, it makes sense…it's a natural thing. I knew it was going to happen but just knowing it had happened…that it was real in a way… I think it's the only time it has affected me this way, because … I already knew that it was part of the job…I knew what it involved [takes a deep breath]. Therefore, I somehow ended up with a natural view of what death is and what it means…that it is a common thing…a part of life, part of nature; this is how it should be. I think that's sort of why I have the view of death that I have.

However, although the participants see death as an expected and natural part of life, a resident's death can also come as a surprise.

#### Subtheme: “Have I failed?”

Several participants describe feeling “shocked” when a resident they felt close to died unexpectedly. Milana felt taken by surprise when a resident (nearly a centenarian) had died:

It went very fast, poof—and then she was gone. She had a heart attack while sitting…and it was painful…It's very special when someone dies suddenly and unexpectedly. You’re not ready for it…you’re on your way home when your colleague brings a sad message…‘you know what… your favorite is gone.’ Then you think how?…She was just here, had just eaten porridge.

Although participants know that residents will eventually die in their care, they can still experience a sense of failure when a resident they feel close to dies suddenly. For example, Timmy talks about a resident with whom he had a close relationship. When the resident died, he “felt bad”:

I was at work the day before he died, I remember, and when I went home from work, he did not seem to be unwell…but he died the next day. In this case I felt very…why?…What happened?…Have we done anything wrong?…Have we not done what we were supposed to do for him? I remember well that he had no signs that he was going to die when I went home from work, so… it was a bit ‘oh gosh’ for me.

For participants, making sense of a resident's sudden death was difficult when they felt concerned about not having done “the right thing” or not having “done enough.”

#### Subtheme: “It's the Best Thing for the Residents”

Participants try to make sense of a resident's death, for example by saying that “it’s the best thing,” reflecting a shared understanding that when a resident is suffering, death can bring relief and peace:

I see so much pain, so much discomfort … people who have pressure sores for many years…sores that never heal…and it's not nice to see…I…it might be strong words, but I consider it as torture…at least for some…I know I myself had considered it as torture…just being kept in a bed…maybe get a visit once a year…totally isolated from family and friends and … yes … no … I think it's very undignified. (Even)

Here we can see how strongly Even immerses himself in the residents’ predicaments. When he perceives a resident's situation as painful and isolated, he places himself into the other's shoes, identifying with the resident as he considers this predicament as undignified torture.

Other participants rely on a religious outlook on the afterlife to make sense of death. For example, Timmy finds comfort in the belief that the residents are going to “a better place”:

When they die, it's about…it's both painful and good, I can say, because when they die, they don't suffer anymore…now they are at peace. Perhaps it's because I am religious, we know where they go after that…yes, since they are also old, I think… yes, that's the best thing for them.

In contrast, Karina is not so sure that it is best for the residents to “let go”:

In my religion, it is not a given that everyone who dies goes to heaven, you have to work a little to achieve it…Therefore, I cannot say one hundred percent if it was best for him or her to die.

Regardless of different views on the possibility of an afterlife, participants share a concern with saying goodbye to dying residents.

#### Subtheme: “It's Important to say Goodbye”

Participants underline the importance of “saying goodbye” to a resident they feel close to in order to achieve “closure.” Being unable to do so can arouse difficult feelings. Here Timmy talks about a special resident:

I remember he was taken down to the cold room very quickly. I didn't get the chance to see him…So, I felt a bit like…bad in a way…because oh… I didn't even get to offer my condolences…speak to the family a last time…I think it's important…to finish…as a final phase in a way… …it's important to have a closure. (Timmy)

Here, Even reflects more generally on the kind of experience Timmy narrates above, suggesting that partaking in rituals could be of help to gain a sense of closure after a resident's death:

It's a person you’ve known for a long time, and of course you’ve had a relationship with them…and there is no one else in this world for whom you would not have attended their funeral, if you’d met them so often. Because you see them more often than your best friends… more often than you see your family too. I think it [attending the funeral] could have been good, just for the closure. Perhaps we need a tradition here…some kind of memorial thing for residents. Could have been nice.

Participants reflected on the disappointment of not being able to say goodbye or even offer condolences to next of kin, thereby expressing their own need for closure and social conventions, e.g. memorial rituals, whether outside or inside the institutional context.

### Superordinate Theme 3: Struggling to Balance Between Being Personal and Professional

#### Subtheme: “It's Not Private, you Mustn't Take it Home, but you are a Human Being”

Many of the participants grapple with the idea that emotions they experience in relation to the residents belong in the private sphere and not to their professional caring practice. They mourn the loss of the relationship they had with the resident, yet, they reflect an attitude that such feelings are not compatible with being professional. When “a special resident” dies, several of the participants see no other option but to just go on with their job as usual. Due to patient confidentiality, participants find it difficult to talk about their emotional experiences even at home. Here Milana talks about the struggle to balance between work and privacy:

You almost sit in grief; you have no energy…You think about it…no matter what…it's inside you…and since you have a duty of confidentiality you can't talk about it…Everything is going through us…diseases and deaths…everything. Nevertheless, eventually you get used to it…you put it behind…what is at work must be at work; at least you have to learn not to take it home. It's not private; you must not take it home. But you are a human being… of course you’re sitting with your thoughts at the dinner table… ‘oh my God… it happened to her’… You do.

Milana appears to feel suffused by the experience of residents’ diseases and deaths (“everything”) and puts it behind her by separating professional experiences from her private sphere. Yet this attempt “not to take it home” appears futile, as certain thoughts and feelings cannot be warded off—because she is a human being, as well as being a professional.

#### Subtheme: “I Have to Learn to Survive”

Milana is not alone. Other participants too try to protect themselves from potential grief by defining clear boundaries between work and private life. Karina describes her strategy:

Usually I cut the job as soon as I leave [the nursing home]. This I manage to do…but it doesn't stop me from being fond of the residents. I’ve learned to do this because I have to survive. I have a life; I have my parents, who are old. … I have to learn to survive and I have learned it through those years.

For Karina, a clear separation between working life and private life is a matter of “surviving” but her emphasis on “learning” suggests that this strategy may not have been her default mode as she entered the NH. Even appears to protect himself from becoming emotionally attached to residents, and the boundary between work and home is embodied and ritualized in his act of taking on and off his uniform:

In order to protect myself, I have at least chosen to… yes… to put the feelings a little on the side. I’m not emotionally invested in the residents. Moreover, I think that's an advantage because I notice that…on such sad days [when a resident has died] I can…As the uniform goes off, so does the feelings. When I go home, regardless of what that day offers, I am 100% myself. Then the caregiver will remain at the ward, and as soon as I put my clothes on again, I will be back. When the uniform goes off, the person goes home and the caregiver stays.

Participants share the notion that it is a professional ideal (and for Even, a ritualized practice) to leave emotions and experiences at work behind and not bring them into private life, but reveal how this is actualized with varying degrees of “success.”

#### Subtheme: “In the end, They are Part of our Lives in a way”

The prevalence of strong bonds becomes evident when participants recall dead residents, even after many years. “I think about them from time to time, all the way back to the time when I was a nursing assistant, because they stay with you,” Even says—in stark contrast to what he said earlier about not being emotionally invested in the residents. Many of the participants have specific memories of how the residents “talked” and they remember their “stories,” illustrated by Karina here:

There are people who died 15 years ago, and I remember them well. Sometimes I drive past their house and…. I have them with me, everyone who has lived here [at the nursing home] for a while; I remember relatives, remember episodes, good and bad and learn from them. In the end, they are part of our lives in a way.

Reidun recalls a unique resident and their special relationship: “I just keep him deep in my heart … yes, he's there … so it's an experience to take on board … how attached we can be to the residents.”

## Discussion

The aim of this study was to explore and understand NH staff's emotional experiences of being in a close relationship with a long-term resident who died. Across the six cases analyzed, we found, perhaps not surprisingly [to readers familiar with this field], that staff care deeply for some residents; that they engage in and treasure such relationships, and that they strive to make sense of the resident's death. Illustrative of this is the way in which they talked about their “special residents,” described in terms such as “being fond of” and “feeling close to.” In line with other studies (Canham et al., 2017; Moss et al., 2003) we found that participants often viewed their relationships with special residents as familial, reminiscent of their own relatives. Their emotional engagement in caring relationships with residents was also revealed by their wish to say goodbye to the dying one last time or to attend the funeral of the deceased. Consistent with previous studies (Boerner et al., 2015; Cagle et al., 2017; Marcella & Kelley, 2015) we found that NH staff grieve when residents they feel close to die, and that they retain memories of such special deceased residents for many years.

### Existential Responsibility

The participants wanted to do good for and be good to special residents with whom they had a close relationship and gave a lot of themselves. They conveyed something deeply satisfying about caring for these residents. However, they also experienced significant emotional pressure associated with the risk of doing something that is wrong or not good, wondering whether they had failed as professionals. The residents’ vulnerability, dependence, and needs can present themselves in an “ethical demand” (Løgstrup, 1997), that may be both silent and unfillable in nature. Based on their understanding of the demand, professionals may feel called (rather than commanded) to act, holding a vital part of the resident's life in their hands, relationally and existentially. But an ethical demand also invokes relational interdependence and mutual vulnerability, as is evident in our participants’ expressed self-doubt and concerns about having done something wrong or not enough. This is consistent with a previous study (Karlsson et al., 2017), which found that existential responsibility can evoke feelings of guilt, failure, and self-doubt in the nurses caring for patients at the end of life. Yalom (1980) refers to responsibility as existential given, correlating with freedom. In this sense, freedom is rooted in an awareness of one's perceived responsibility to create one's world. He describes this insight as deeply frightening because it means one is responsible not only for one's own successes but also for one's failures. In our study, responsibility in the response to the ethical demand was accompanied by staff's anxious feelings of not “doing the right thing” or “doing enough” for the residents. The irreversible aspect of death may reinforce the participants’ sense of responsibility, as well as their anxiety or guilt when caring for long-term residents who are often close to death—as they only have one chance to do it right and cannot correct mistakes later.

### Mutual Recognition

We have thus far brought ethical demand and existential responsibility into our interpretation of NH staff's close relationships with special residents in long-term care. Such existential responsibilities, which are inherent in any close relationship, can be further interpreted as anxiety provoking, posing a threat to the professional's identity. This may go some way to account for their splitting and ambivalence about the personal and professional as psychological defenses against anxiety (Ramvi & Gripsrud, 2017). But alongside these threats and anxieties, are the participants’ deeply meaningful experiences of being a significant other to a resident. Because NH staff know that most long-term residents will eventually die in their care, to feel recognized in the caring relationship, by residents and their family, gains significance. Of note is how participants’ experiences with a close relationship strongly reflected the professional's *own need* for feeling recognition and appreciation in the relation with the deceased residents and their relatives. We can understand this as an expression of human interrelatedness and mutual dependency, aspects of which may resonate or interrupt in any professional relationships (Ramvi, 2011). Benjamin (2004, p. 5) defines such intersubjectivity in terms of “a relationship of mutual recognition” in which “each person experiences the other as a ‘like subject,’ another mind who can be ‘felt with,’ yet has a distinct, separate center of feeling and perception.” Although our participants’ focus in long-term care is on the resident and next-of-kin, their experiences reveal how such work may also entail a profound sense of reciprocity when professionals become engaged in close caring relationships. Illustrative of this is Even who immerses himself in the residents’ lives. He empathizes with them in the sense of feeling with them and thinking about what it would be like if it were him facing the same predicament as the resident.

In recalling and recounting past relations, participants emphasized their own needs or wish to feel recognized, appreciated, confirmed, accepted, and tolerated for who they are as a person. This is evident in Reidun's narrative where she talks about how positive feedbacks from residents and their families make her feel good, both as a nurse and as a person. Perhaps this was especially so because the participants felt an existential responsibility for residents who were at the end of life. Following our interpretative strands thus far, we could say that it was imperative for them, personally and professionally, to do a good job in response to perceived existential responsibility and ethical demands, because they felt intersubjectively interpellated (a “relational calling”) in close relationships with special residents.

### Struggling to Balance the Personal in the Professional

A striking finding was how participants struggled to balance the personal and professional in their close relationships with residents who later died. In line with previous research (Moen, 2018; Ramvi, 2011) they experienced tensions between established ideals of professionalism on the one hand, and on the other hand, personal experiences of deep commitment and strong emotions in the relationships with their “special residents,” illustrated by Even who sought to uphold distance but found himself bonding with a resident, nevertheless. Health care staff are expected and obliged to be professional in caring for long-term residents. However, as numerous authors have pointed out, open communication about personal experiences with death in whole person care can be viewed as *integral* to professional practice and not as opposed to it (see e.g. Ramvi & Gripsrud, 2017). Nyatanga (2012) recognizes that professionals need to be comfortable talking about death themselves to facilitate care and communication for residents and their next of kin. Participants in our study appeared divided or ambivalent on this topic. A particular concern for them was the management of emotional experiences of residents’ deaths that could potentially accompany them into private life. Interestingly, the NHs did not appear to accommodate staff's processing of emotional experiences at work. In fact, the fieldwork observations of the first author suggested an institutional practice or culture of silence. None of the NHs offered staff systematic provisions for talking about relational experiences with residents or with residents’ deaths. Institutional norms and ideals of rationalizing professionalism may offer emotional distance to protect staff from being confronted with challenging emotions at work, but according to Funk et al. (2017) and Gripsrud et al. (2020), this may not be the best strategy. In what follows, we therefore wish to take a closer look at staff's experiences of personal commitment and what makes these relational experiences so emotionally powerful—and why these should be a topic of conversation in the NH.

### Divergent Discourses of Professionalism

As we have seen, participants’ experiences of being in a close relationship with residents in long-term care are both deeply meaningful, rewarding and anxiety inducing. We have suggested that existential responsibility and mutual recognition emerge due to the intersubjective nature of their relational work. However, personal feelings can appear incompatible with attitudes and ideals about professionalism. In what follows, we pursue this conundrum through a discussion of tensions between discourses of professionalism and personal experiences rooted in intersubjectivity.

Balancing tensions between being personally involved and emotionally distanced has been identified as particularly salient in work with persons at the end of life and their relatives. In a previous ethnographic study, Candrian (2014, p. 53) identified how caregivers working at a hospice and an emergency department “tamed death” through linguistic practices to reduce the complexity and overwhelming emotional challenges that accompanied their work, thereby disconnecting from their own experiences. Simard’s (2020) recent ethnographic study of caregiver suffering in a hospice, identified two types of positions toward the establishment of therapeutic relationships. Firstly, there was the “official position” in hospice care of a therapeutic relationship that promotes an idealized, symbiotic relationship with patients and relatives, where professionals allow themselves to be emotionally engaged (Simard, 2020, p. 8), in line with the intersubjective perspective we presented above. Secondly, and in contrast to the official position, was the “reality position” of professionals who were searching for an appropriate distance so as to protect themselves from a psychological strain. In our study, we also find evidence of both discourses. Our findings, emerging from fieldwork and interviews, indicate that there is taming of death in a shared expectation of “professionalism.” According to this notion of professionalism, staff should not become emotionally involved when caring for residents and emotional experiences could not be talked about at work. Death could be normalized, reflected in standard phrases such as “death is a natural part of life” and “death is to be expected in the nursing home”—where participants viewed death as a “friend and liberator” (Ådland et al., 2019). Observations of this taming, discursive practice in the two NHs may indicate the presence of a collective social defense against anxieties and vulnerabilities induced by relational work (Menzies-Lyth, 1959/1988).

Participants also expressed a more personal ideal of a caring and therapeutic relationship—of being close with “special residents,” almost like family, in line with Simard’s (2020) findings. By being emotionally engaged and “making a difference” in a resident’s life, they experienced a deep sense of reward when they were able to do something good, illustrated by Timmy's narrative. To understand how our findings touch on Candrian (2014), and Simard (2020), we suggest the following line of interpretation: although the accumulated intensity of feelings produced by close relationships in long-term care can be said to reflect a certain level of intimacy for some participants, for others it may be an underlying contributor to their desire or need to distance themselves from becoming emotionally involved. The wish to remove themselves from emotional reality or engagement may be “disguised” in the discourse of professionalism, where the ideal is to “tame death” by disconnecting work and personal experience. In contrast to this discourse, lies the staff's potential for fully experiencing the reality of a “special relationship” *and* engaging with the existential responsibility it entails, which may offer a sense of fulfillment and meaningfulness, even long after the resident's death.

## Implications for Holistic Nursing

Holistic nursing emphasizes the importance of establishing relationships between care givers and care receivers. Such relationships can be both enriching and challenging for health care professionals who work in long-term NH care, where residents’ deaths are commonplace. When close relationships are established with special residents, professionals use themselves in a personal way. This form of professional–personal dedication can exacerbate a sense of vulnerability and culpability when a resident with whom they have a close relationship dies. Suppressing such challenging emotions associated with residents’ deaths puts health care professionals at risk of developing burnout or compassion fatigue.

To support sound professional practice and staff's wellbeing in NHs, there should be more awareness of challenges and rewards associated with working in long-term care. Based on our findings, we make the following suggestions.

Firstly, NH managers should systematically initiate discussions in the staff community on ideals surrounding “distanced professionalism” versus ideals corresponding with caring relationships. Through such facilitated discussions, NH staff can be supported to make sense of, reflect on and process their experiences and thereby develop their professional identity. Moreover, these facilitated discussions can allow for the articulation of professionals’ experiences of close relationships with residents, which may evoke difficult feelings of vulnerability or culpability. The organizational culture of NHs can be enriched by introducing such reflective practices that can help staff accommodate and recognize that in relationship-based whole person care, the personal is an indelible part of being a professional. This is especially so in long-term NH care, which, for health care professionals, involves repeated emotional experiences of establishing contact, bonding, and caring, while also dealing with separation and grief. Secondly, management should encourage professionals to partake in rituals, such as attending a resident's funeral, and/or establishing an institutional memorial to mark the occasion of a resident's death. Rituals are simple measures that provide comfort and can help contain professionals’ emotional distress.

## Conclusion

NH staff experience close personal relationships with residents that may arouse strong feelings. The experience of such emotional bonds can contribute positively to a caring relationship by evoking an existential responsibility. But such experiences can also evoke strategies of distancing and separation for NH staff. We suggest that the unconscious need or desire to distance or separate oneself from emotional experiences may be “disguised” in a discourse of professionalism, which concerns the ideal of the distanced and emotionally detached professional. Our findings show that NH staff are confronted with ethical demands and existential responsibility in caring for residents in long-term care facilities. Receiving recognition in a mutually constitutive relation with residents is a profoundly intersubjective experience, which may allow staff to bear “death at work” (Moen, 2018), and is at odds with the taming, splitting, and distancing discourse of professionalism. When staff feel that their efforts to do good and be good in close caring relationships are recognized, acknowledged, and appreciated by the care receivers—*and* when personal and emotional experiences can be recognized as integral to their professional mindset—it can provide the vigor, fulfillment, and meaningfulness needed to sustain demanding work in caring relationships with residents in long-term care.
